# Rb induces a proliferative arrest and curtails *Brn-2 *expression in retinoblastoma cells

**DOI:** 10.1186/1476-4598-5-72

**Published:** 2006-12-12

**Authors:** David Cobrinik, Richard O Francis, David H Abramson, Thomas C Lee

**Affiliations:** 1 Margaret M. Dyson Vision Research Institute, Weill Medical College of Cornell University, New York, NY 10021, USA; 2 Department of Ophthalmology, Weill Medical College of Cornell University, New York, NY 10021, USA; 3 Department of Pathology, Columbia University College of Physicians and Surgeons, New York, NY 10032, USA; 4 Ophthalmic Oncology Service, Memorial Sloan-Kettering Cancer Center, New York, NY 10021, USA; 5 Division of Ophthalmology, Department of Surgery, Childrens Hospital Los Angeles, Los Angeles, CA, USA

## Abstract

**Background:**

Retinoblastoma is caused by loss of the Rb protein in early retinal cells. Although numerous Rb functions have been identified, Rb effects that specifically relate to the suppression of retinoblastoma have not been defined.

**Results:**

In this study, we examined the effects of restoring Rb to Y79 retinoblastoma cells, using novel retroviral and lentiviral vectors that co-express green fluorescent protein (GFP). The lentiviral vector permitted transduction with sufficient efficiency to perform biochemical analyses. Wild type Rb (Rb^WT^) and to a lesser extent the low penetrance mutant Rb^661W ^induced a G0/G1 arrest associated with induction of p27^KIP1 ^and repression of *cyclin E1 *and *cyclin E2*. Microarray analyses revealed that in addition to down-regulating E2F-responsive genes, Rb repressed expression of *Brn-2 *(*POU3F2*), which is implicated as an important transcriptional regulator in retinal progenitor cells and other neuroendocrine cell types. The repression of *Brn-2 *was a specific Rb effect, as ectopic p27 induced a G0/G1 block, but enhanced, rather than repressed, *Brn-2 *expression.

**Conclusion:**

In addition to Rb effects that occur in many cell types, Rb regulates a gene that selectively governs the behavior of late retinal progenitors and related cells.

## Background

Retinoblastomas form due to the inactivation of the *RB1 *gene together with other genetic changes [1]. Whereas *RB1 *is also inactivated in other tumors, the cells that give rise to retinoblastoma are exceptionally sensitive to Rb loss. Individuals with bilateral retinoblastoma (and presumed germ line *RB1 *mutations) develop an average of five retinoblastoma foci, generally within their first 2 years [2,3], but have only ~1% per year likelihood of developing all other tumor types [4]. Moreover, retinoblastoma is largely a human-specific disease. Tumors with histopathological features of retinoblastoma, including photoreceptor but not amacrine differentiation, have been reported for only two individual animals [5,6] and appear not to form in response to the loss of Rb family proteins in mice [7-10].

Rb has numerous functions that might mediate the suppression of retinoblastoma [11]. Studies in non-retinal cells have shown that Rb can inhibit cell cycle progression through regulation of E2F1 and p27^Kip1 ^[12-14], and may induce a senescence-like response through E2F-dependent or E2F-independent mechanisms [15-17]. In addition, Rb promotes differentiation through interactions with several widely expressed proteins [18,19], and may both promote differentiation and suppress tumorigenesis by inhibiting Ras [20]. Besides these general effects, Rb promotes osteogenic, adipogenic, thyroid, and melanocytic differentiation through interactions that are specific to the relevant cell types [21-26]. Thus, to understand how Rb suppresses retinoblastoma, it may be necessary to identify the cell type-specific functions of Rb in the cells that give rise to retinoblastoma tumors.

At present, the cell type that gives rise to retinoblastoma has not been identified. It was proposed that retinoblastomas arise from a primitive neuroectodermal cell [27], such as a retinal progenitor cell (RPC) or a transition cell that fails to arrest in Rb's absence during early differentiation [28]. More recent evidence suggests a possible origin from post-mitotic cone precursors [29]. However, defining Rb's tumor suppressor role in any of these cell types is problematic, as human retinal cells are poorly suited to growth and manipulation *in vitro*, and as Rb's tumor suppressor role may not be replicated *in vivo*, in mouse models. As an alternative to working with the retinoblastoma cell of origin, insight into Rb's role may be gained by examining the effects of restoring Rb to retinoblastoma cells. While signaling in such cells may differ to some extent from that in the cell of origin, cell type-specific retinoblastoma suppressor functions of Rb seem more likely to be manifested in retinoblastoma cells than in other available cell types.

The effect of restoring Rb to retinoblastoma cells was first examined soon after the *RB1 *gene was cloned. When Rb was restored using Murine leukemia virus (MLV)-based retroviral vectors, transduced cells were analyzed after >4 weeks of selection and displayed either modestly diminished proliferation [30,31] or no obvious change in proliferative rate [32-35]. In contrast, when Rb was restored by transfer of chromosome 13 (on which *RB1 *resides), proliferation was clearly impaired [36]. This suggested that expression of Rb under its normal regulatory sequences conferred a stronger antiproliferative effect than was conferred with MLV-based vectors. However, chromosome transfer is too inefficient to be used to define Rb's acute effects, and cannot be used to compare effects of wild type and mutant Rb proteins.

To define the effects of restoring Rb to retinoblastoma cells, we developed retroviral and lentiviral vectors that co-express Rb and enhanced green fluorescent protein (GFP). As MLV-based retroviral vectors have a propensity to be silenced by trans-acting factors and DNA methylation, we used murine stem cell virus (MSCV) and lentivirus vectors that have diminished silencing [37,38]. Use of the GFP marker permitted the acute effects of Rb to be observed in the absence of antibiotic selection of transduced cells, and permitted the efficiency of transduction to be determined prior to biochemical analysis. These studies demonstrate that several of the Rb effects that have been observed in other cell types are also manifested in retinoblastoma cells. In addition, they identify an Rb-regulated gene that may have a crucial role in retinal cell proliferation.

## Results

### Restoration of Rb to Y79 cells using the MSCV-GFP retroviral vector

**Figure 1:**
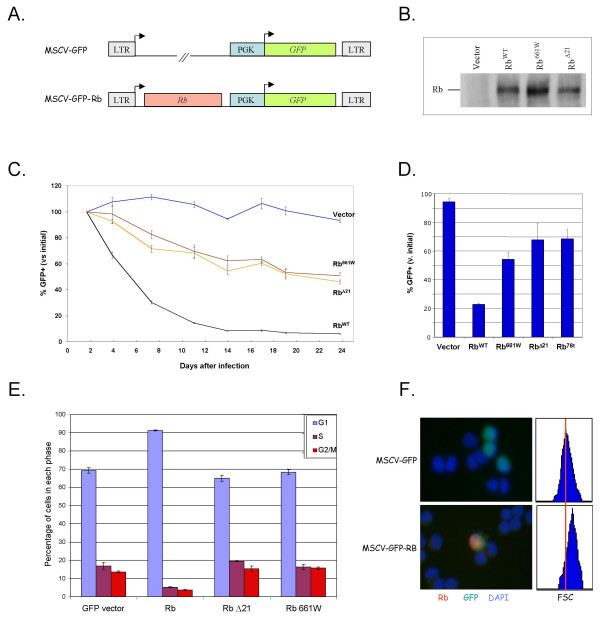
**Effect of retroviral Rb transduction on Y79 proliferation**. **A**. Structure of the MSCV-GFP retroviral vector and Rb derivatives. Rb is expressed from the MSCV LTR, and GFP from a PGK promoter. **B**. Western analysis of Rb expression 3 days after infection. **C, D**. Prevalence of GFP+ cells after infection with the indicated vectors, relative to that at 44 h after infection, displayed as a time course (C) or at day 12 for a separate experiment (D). **E**. Effect of Rb on cell cycle distribution 84 h after infection. **F**. Effect of Rb on cell size. Cells infected with MSCV-GFP or MSCV-GFP-Rb were identified by GFP fluorescence and stained for Rb (left) or measured for forward scatter (FSC) by flow cytometry (right). For C-E, infections were in triplicate, data points represent averages, and error bars indicate standard deviation.

In initial experiments, Y79 cells were transduced with the MSCV-GFP vector or derivatives encoding wild type or mutant Rb (Figure 1A). In these constructs, Rb was expressed from the MSCV LTR and GFP from a PGK promoter 3' to the *RB1 *cDNA. The versions of Rb examined included the wild type (Rb^WT^), the tumor-derived mutants Rb^Δ21 ^and Rb^Δ22 ^[39], a low penetrance mutant (Rb^661W^) that retains partial tumor suppressor function but lacks the ability to regulate E2F1 [40], and a mutant (Rb^76t^) with a premature termination codon at amino acid 76 [41].

Viral supernatants were combined with Y79 cells, and infection monitored by flow cytometry. GFP+ cells were first detected ~30 h after infection, and peaked at 44 h, typically in 10–20% of cells. At three days after infection, western blotting indicated that similar levels of Rb^WT ^and Rb^Δ21^, and slightly higher levels of Rb^661W^, were produced (Figure 1B).

### Antiproliferative effect of retrovirus-transduced Rb

To determine whether restoring Rb with the MSCV-GFP vector affected Y79 proliferation, the percentage of GFP+ cells was determined at various times after infection and normalized to that at 44 h (Figure 1C). The proportion of GFP+ cells remained constant in cultures transduced with MSCV-GFP, indicating that transduced and untransduced cells proliferated at similar rates. In contrast, the proportion of GFP+ cells rapidly declined after transduction with MSCV-GFP-Rb^WT^, suggesting that proliferation of Rb-transduced cells was impaired.

Cultures that were transduced with the low penetrance mutant Rb^661W ^and the tumor-derived mutants Rb^Δ21 ^and Rb^Δ22 ^displayed an intermediate decline in GFP+ cells (Figure 1C and data not shown). To determine whether this decline required expression of mutant Rb protein, Y79 cells were transduced with MSCV-GFP-Rb^76t^, which encodes a truncated and apparently unstable product [41]. As Rb^76t^-transduced cultures displayed a similar decline in GFP+ cells (Figure 1D), the decline observed with each mutant was likely due to a *cis *effect of *RB1 *sequences that resulted in silencing of *GFP *expression.

To better define the anti-proliferative effect of Rb^WT^, transduced cultures were stained with propidium iodide and the cell cycle profile of GFP+ cells examined. Rb^WT^, but not Rb^661W ^or Rb^Δ21^, increased the proportion of GFP+ cells in G0/G1 and reduced the proportions in S and G2/M (Figure 1E). To our knowledge, this is the first demonstration that restoration of Rb alters the cell cycle profile of retinoblastoma cells. Rb^WT ^but none of the Rb mutants also increased Y79 cell size, as measured by immunocytochemical staining and the flow cytometry forward scatter parameter (Figure 1F and data not shown).

### Culture of Rb-transduced cells selects for low Rb expression

**Figure 2:**
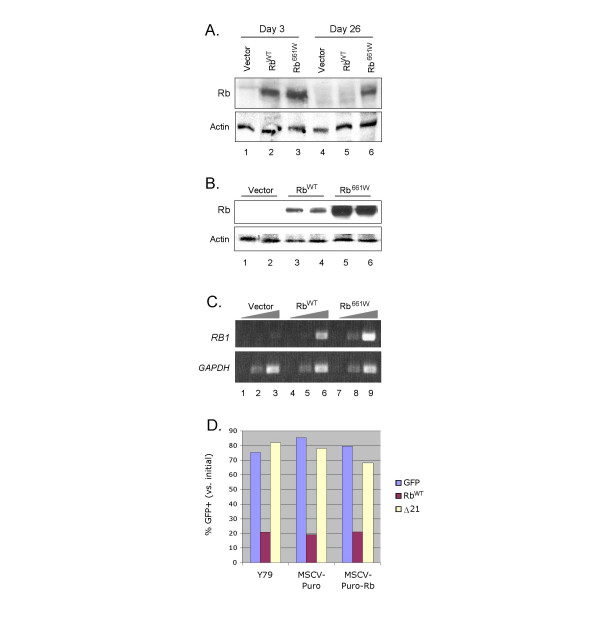
**Long-term culture of Rb-transduced cells selects for low Rb expression**. **A**. Rb expression in Y79 cells 3 days after transduction with MSCV-GFP and Rb derivatives (lanes 1–3), or in puromycin resistant Y79 cells 26 days after transduction with MSCV-Puro and Rb derivatives (lanes 4–6). Lysates from infected cultures were combined with mock-infected cell lysates to normalize the proportion of GFP+ cells. **B**. Rb expression after transduction with the indicated MSCV-Puro viruses and puromycin selection, at 19 days (lanes 1, 3, and 5) and 26 days (lanes 2, 4, and 6) after infection. For **A **and **B**, actin expression is displayed as a loading control. **C**. RT-PCR analysis of *RB1 *and *GAPDH *mRNA in puromycin resistant cells after infection with the indicated viruses, with PCR for 22, 26, and 30 cycles. **D. **Prevalence of GFP+ cells at 12 days relative to that at 44 h after infection of Y79 cells, or after infection of puromycin-selected Y79 derivatives that were earlier infected with MSCV-Puro or MSCV-Puro-Rb.

In cultures that were transduced with MSCV-GFP-Rb^WT^, the proportion of GFP+ cells declined for two weeks, but then remained constant (Figure 1C), indicating that the remaining GFP+ cells proliferated at the same rate as uninfected cells. To determine whether such cells were selected for decreased Rb, we examined Rb expression in cell populations that were equivalent to the GFP+ cells in MSCV-GFP-Rb transduced cultures, but which expressed puromycin resistance instead of the GFP marker. To do so, Y79 cells were infected with the MSCV-Puro vector or derivatives encoding Rb^WT ^or Rb^661W^, and selected with puromycin. After selection, cells that were transduced with MSCV-Puro-Rb^WT^, but not those transduced with MSCV-Puro-Rb^661W^, had markedly reduced Rb protein (Figure 2A, B) and *RB1 *RNA (Figure 2C). Moreover, when the MSCV-Puro-Rb^WT^-transduced and puromycin-selected cells were re-infected with MSCV-GFP-Rb^WT^, they displayed a rapid decline in the proportion of GFP+ cells, similar to that of the parental Y79 and vector-transduced controls (Figure 2D). This confirmed that Y79 cells that proliferate after Rb transduction remain sensitive to Rb, but fail to arrest due to their low Rb levels.

### Restoration of Rb using concentrated retroviral and lentiviral vectors

**Figure 3:**
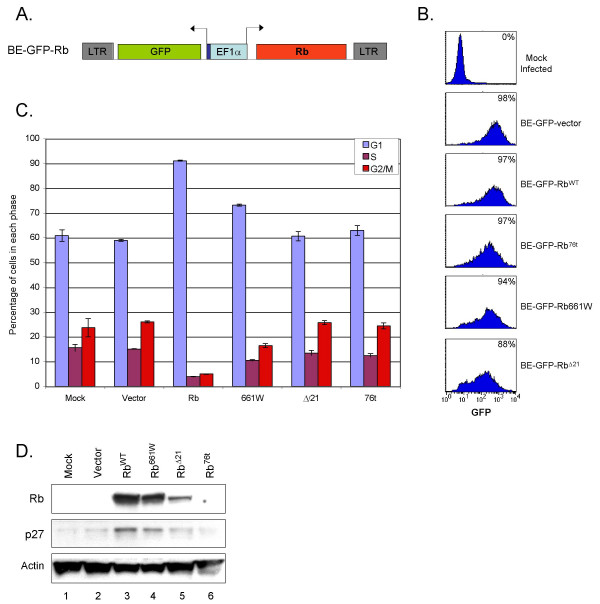
**Lentiviral transduction of Y79 with BE-GFP-Rb**. **A**. Structure of BE-GFP-Rb^WT^. Rb and GFP are expressed from a bidirectional promoter consisting of *EF1α *enhancer + promoter (light blue) and a minimal *CMV *promoter (dark blue) that requires a heterologous enhancer for activity [56]. **B**. Flow cytometric analysis of GFP expression in transduced cultures at 56 h after infection. Numbers indicate the percent GFP+ cells. **C**. Y79 cell cycle profiles 60 h after mock infection or transduction with the indicated BE-GFP viruses. **D**. Western analysis of Rb, p27, and α-actin 60 h after mock infection (lane 1) or infection with the indicated BE-GFP viruses (lanes 2–6).

We next sought to quantitatively restore Rb to Y79 cells, in order to define Rb's biochemical effects. As concentrated MSCV-GFP-Rb preparations infected at most ~40% of Y79 cells, transduced cells were further enriched by FACS, to yield populations that were ≥ 97% GFP+. As a second approach, we developed a lentiviral vector to express Rb and GFP, termed Bidirectional-EF1α-GFP (BE-GFP, Figure 3A). A bidirectional promoter was used to tightly link Rb and GFP expression, and thus minimize production of GFP+ cells that have little or no Rb. Concentrated BE-GFP vector and Rb derivatives were generally of sufficient titer to transduce >94% of Y79 cells (Figure 3B). This permitted analysis of Rb effects without further cell isolation.

### Antiproliferative effect of lentivirus-transduced Rb^WT ^and Rb^661W^

At 60 h after lentiviral transduction, Rb^WT ^induced an accumulation in G0/G1 similar to that induced with retroviral transduction (Figure 3C). Lentiviral transduction of Rb^661W ^also induced an accumulation in G0/G1, though to a lesser extent than Rb^WT^. The ability of Rb^661W ^to elicit a cell cycle block after lentiviral but not retroviral transduction was most likely due to higher levels of Rb^661W ^obtained with the *EF1α *promoter and a higher multiplicity of infection.

**Figure 4:**
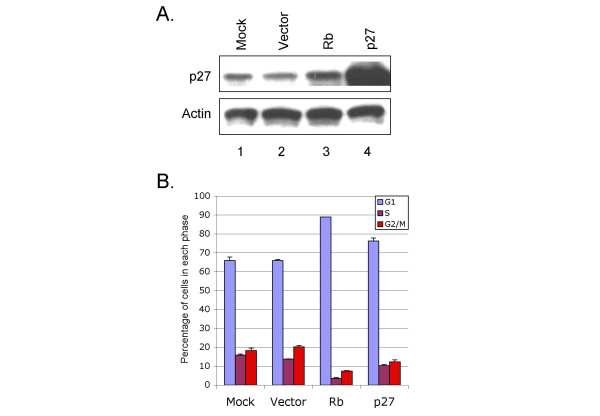
**Comparison of Rb and p27 cell cycle effect**. **A**. Y79 cells were mock infected (lane 1) or infected with BE-GFP, BE-GFP-Rb, or BE-GFP-p27 (lanes 2–4); and examined by western blotting for p27 expression, and for actin expression as a loading control. **B**. Infected cells were stained with propidium iodide and examined for cell cycle position.

Rb^WT ^and Rb^661W ^were previously found to inhibit osteosarcoma cell proliferation by inducing a post-transcriptional increase in p27^KIP1 ^[13,14]. Similarly, Rb^WT ^and Rb^661W ^induced p27 in Y79 cells, at levels that were in proportion to Rb protein expression (Figure 3D). To determine whether p27 might mediate the Rb-induced proliferative arrest, we compared the effects of ectopic Rb and ectopic p27, using the same lentiviral vector to express each protein. Lentiviral p27 transduction resulted in higher p27 levels, but caused less of an accumulation in G0/G1, as compared to lentiviral Rb (Figure 4). This implies that p27 does not fully mediate the Rb-induced G0/G1 arrest.

### Effect of Rb on gene expression

Rb is thought to inhibit proliferation, in part, by repressing E2F-regulated cell cycle genes and promoting expression of cell type-specific differentiation genes. Thus, we performed a microarray analysis to assess Rb's effects on E2F-responsive genes and to identify additional Rb-regulated genes in retinoblastoma cells. RNA was obtained from FACS-purified GFP+ Y79 cells at 60 h after transduction with MSCV-GFP or MSCV-GFP-Rb^WT^, and analyzed by Affymetrix GeneChip. Among well-described E2F-responsive genes, *CDC2*,*CDK2*, *cyclin E2*, *polo-like kinase *(*PLK*), and *p107 *(*RBL1*) were scored as more than 2-fold repressed, whereas others had comparatively little change (Table 1). Of these, *cyclin E2 *and *p107 *are among the small subset of E2F-responsive genes that typically require Rb for normal regulation [11,42-44]. Rb had little effect on *cyclin E1*, despite that Rb is required for normal *cyclin E1 *regulation in other cell types [44,45].

**Table 1:** Gene expression effects of retroviral Rb transduction

**Gene**	**Probe Set Accession #**	**Fold Change**
**Controls**		
*GAPDH*	M33197_M_at	0.92
	M33197_3_at	0.91
	M33197_5_at	0.82
β-actin	200801_x_at	1.07
		
**E2F-responsive**		
*CDC2*	203214_x_at	0.53
	210559_s_at	0.63
	203213_at	0.70
	231534_at	0.43*
*CDC25A*	204695_at	0.60
	1555772_a_at	0.76
*CDC25B*	201853_s_at	0.93
*CDC25C*	205167_s_at	0.89
*CDK2*	204252_at	0.38*
*Cyclin A2*	203418_at	0.61
*Cyclin B1*	214710_s_at	0.74
	228729_at	0.76
*Cyclin B2*	202705_at	0.76
*Cyclin E1*	213523_at	0.92
*Cyclin E2*	211814_s_at	0.37*
	205034_at	0.61
*E2F1*	204947_at	0.92
	2028_s_at	0.67
*E2F2*	228361_at	0.56
*E2F3*	203692_s_at	1.31
	203693_s_at	1.02
*EMI1 *(*FBXO5*)	*218875_s_at*	0.66
	234863_x_at	0.55
*Geminin*	218350_s_at	0.63
*HPRT*	202854_at	0.65
*N-myc*	209757_s_at	1.06
*PCNA*	201202_at	0.90
*PLK*	214372_x_at	0.80
	202240_at	0.42*
*RbL1 *(*p107*)	1555004_a_at	0.44*
*Ribonuc. Reductase M1*	201477_s_at	0.65
	201476_s_at	0.50
*Ribonuc. Reductase M2*	209773_s_at	0.74
*SKP2*	210567_s_at	1.16
	203626_s_at	0.77
*Thymidlyate Synthase*	202589_at	0.74
	1554696_s_at	0.57
*Thymidine Kinase*	243103_at	0.98
	1554408_a_at	0.82
	202338_at	0.59
		
**Novel**		
*Brn-2 (POU3F2*)	207084_at	0.05**

Affymetrix U133 Plus 2.0 microarrays were hybridized with probes from vector- and Rb-transduced cells and analyzed with Affymetrix Microarray Suite software. The indicated probe sets were flagged as "Present" in vector transduced samples, and showed the indicated fold change in signal for Rb-transduced versus vector-transduced samples. Asterisks indicate probe sets with fold change less than 0.5. The double asterisk indicates one of the most strongly down-regulated genes in the microarray analysis.

**Figure 5:**
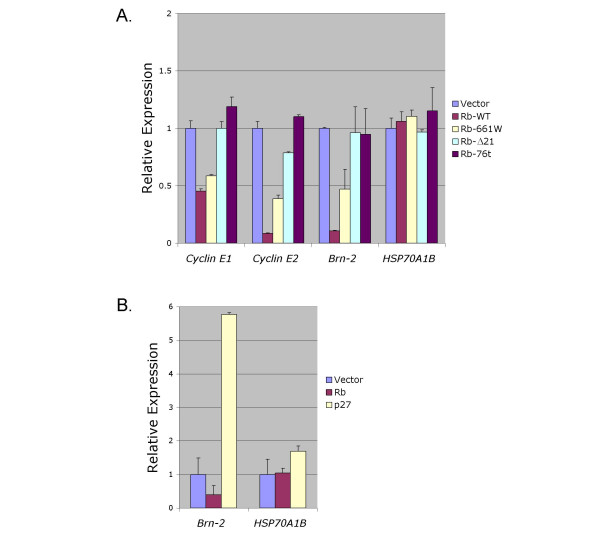
**Gene expression effects of Rb and p27**. cDNA was prepared from cells at 60 h after infection with the BE-GFP vector and **A) **the indicated Rb derivatives, or **B) **either Rb^WT ^or p27 derivatives. The cDNA was subjected to qRT-PCR for the indicated genes. Expression levels were normalized to β-actin and are displayed relative to that of vector-transduced samples. Values indicate averages of duplicate analyses and error bars indicate standard deviation.

We further examined the gene expression effects of Rb and Rb mutants at 60 h after transduction with the BE-GFP lentiviral vector. In quantitative reverse transcription (qRT)-PCR analyses, BE-GFP-Rb^WT ^diminished *cyclin E2 *expression by ~90% and decreased *cyclin E1 *by ~50%, whereas Rb^Δ21 ^and Rb^76t ^derivatives had little or no effect (Figure 5A). The greater effect of Rb^WT ^on *cyclin E *expression after lentiviral transduction and qRT-PCR analysis, compared to retroviral transduction and microarray analysis, may be due to the higher Rb levels expressed from the BE-GFP vector as well as methodological differences. BE-GFP-Rb^661W ^also down regulated *cyclin E1 *and *cyclin E2 *by ~50%. The Rb^WT ^and Rb^661W ^effects appeared to be specific, as Rb did not repress the control gene, *HSP70A1B*.

We next sought to identify additional Rb-regulated genes. One of the most strongly down-regulated genes in the microarray analysis was *Brn-2 *(*POU3F2*), with a ~20-fold reduction in signal (Table 1). This effect was confirmed with qRT-PCR analysis of lentivirus-transduced cells, in which Rb^WT ^repressed *Brn-2 *by ~90% (Figure 5A). Moreover, Rb^661W ^down-regulated *Brn-2 *by 50%, while Rb^Δ21 ^and Rb^76t ^had no effect. Rb's regulation of *Brn-2 *was of interest because *Brn-2 *functions as an oncogene in melanoma cells and is implicated in the control of *Chx10 *and other genes that are expressed in RPCs [46,47]. To determine whether this effect indirectly resulted from Rb-induced cell cycle arrest or p27 expression, we examined *Brn-2 *expression after p27 transduction. Ectopic p27 increased the proportion of cells in G0/G1 (Figure 4B), but induced rather than diminished *Brn-2 *expression (Figure 5B). This implies that Rb does not repress *Brn-2 *through its induction of p27, nor by inducing a G0/G1 arrest.

In summary, Rb not only down regulated E2F-responsive genes, which are thought to have general, cell type-independent functions. Rb also down regulated *Brn-2*, which is particularly important to the regulation of gene expression in RPCs.

## Discussion

Retinoblastoma has long served as a paradigm for cancers that develop due to the loss of a tumor suppressor protein [48]. However, despite that the Rb tumor suppressor was identified 20 years ago, the means by which Rb suppresses this tumor have not been established. In the current study, we examined the effect of restoring Rb to retinoblastoma cells. Our rationale was that Rb might perform functions in retinoblastoma cells that it does not display in other available cell types, and which may relate to Rb's role in the retinoblastoma cell of origin. As the retinoblastoma cell of origin has not been identified, and cell types that are candidates for the cell of origin are not easily manipulated, retinoblastoma cells may serve as the best available setting in which to detect Rb functions that specifically relate to the suppression of this tumor.

Earlier attempts to define Rb's effects in retinoblastoma cells may have been hampered by an inability to efficiently express Rb and thus examine its acute effects. The current study surmounted this obstacle by marking the Rb expression vectors with GFP. This approach revealed that Rb inhibited proliferation, induced a G0/G1 arrest, and increased cell size, similar to Rb's effects in other cell types. Moreover, the studies showed that Rb's anti-proliferative effect was masked when analyzed several weeks after infection, due to the outgrowth of cells that had low Rb levels. This implies that prior studies that failed to demonstrate an antiproliferative effect of Rb after a selection period may have analyzed cells that had ineffective levels or Rb expression. The firm demonstration that Rb inhibits retinoblastoma proliferation validates efforts to restore Rb function as a therapeutic approach [49]. However, our results also revealed that a threshold level of Rb was required to inhibit Y79 proliferation *in vitro*. Whether a similar Rb level is needed to inhibit growth of retinoblastoma tumors *in vivo *remains to be established.

The Rb-induced G0/G1 arrest was associated with increased expression of p27 and decreased expression of E2F-responsive genes, as previously observed in other cell types. In osteosarcoma cells, the induction of p27 preceded the decline in proteins encoded by E2F-responsive genes, and was needed for Rb to rapidly induce a cell cycle block [14]. To date, it has not been possible to determine whether the up-regulation of p27 is required for Rb to arrest Y79 cells, as p27 knockdown did not preclude the induction of p27 by Rb (data not shown). However, our finding that Rb was more effective than p27 in inducing a G0/G1 arrest suggests that Rb inhibits Y79 proliferation at least in part through a p27-independent process, such as by repressing *cyclin E2 *and other E2F-responsive genes. Notably, the low penetrance mutant Rb^661W ^also induced a G0/G1 block, p27 expression, and down-regulation of *cyclin E*, albeit to a lesser extent than Rb^WT^. This is consistent with the ability of Rb^661W ^to induce a proliferative arrest via p27 in osteosarcoma cells [14], and with evidence that Rb^661W ^may regulate E2F-responsive genes through an interaction with E2F2 [50].

In addition to Rb's effect on E2F-regulated genes, Rb strongly repressed *Brn-2*. This effect is notable in light of evidence that *Brn-2 *is highly expressed small cell lung cancers [51], which like retinoblastomas generally lack Rb [39], and in light of evidence that *Brn-2 *functions as an oncogene in melanoma cells [46,52]. Thus, the finding that Rb represses *Brn-2 *raises the possibility that deregulation of *Brn-2 *contributes to retinoblastoma tumorigenesis.

During retinal development, *Brn-2 *is expressed in intermediate and late RPCs as well as in certain post-mitotic cells [47]. Brn-2 may have a widespread transcriptional role in RPCs, as it binds the promoters of the characteristic RPC genes, *Chx10 *and *Nestin*, and may bind related promoter sites in *Cyclin D1 *and *Pax6 *[47]. In cortical development, *Brn-2 *is similarly expressed in late progenitors, and is required in combination with *Brn-1 *for late progenitor cell proliferation [53]. These findings suggest that *Brn-2 *may promote the expression of genes that maintain late progenitor cell proliferation, and that deregulation of *Brn-2 *in response to Rb loss may elicit aberrant expression of such genes, increased proliferation, and retinoblastoma tumorigenesis. Alternatively, as Rb is also expressed in post-mitotic retinal cells [29], Rb-mediated repression of *Brn-2 *may contribute to the suppression of retinoblastoma in post-mitotic retinal precursors.

## Conclusion

Rb was expressed in Y79 cells using novel retroviral and lentiviral vectors. Rb induced a G0/G1 arrest, expression of p27^KIP1^, and repression of E2F-responsive genes such as *cyclin E1 *and *cyclin E2*, similar to Rb's effects in other cell types. In addition, Rb decreased expression of *Brn-2*, which selectively regulates gene expression in RPCs and related cells. Thus, in addition to Rb's cell type-independent effects, Rb regulates genes that control transcription in the developing retina.

## Methods

### DNA constructs

MSCV-GFP was constructed by replacing the HindIII-ClaI fragment of MSCV-Puro (Clontech) with a HindIII-NotI fragment from pEGFP-N2 (Clontech). To produce MSCV-GFP derivatives, BamHI-StuI fragments from pSVE-hRB^WT^, pSVE-hRB^Δ22 ^[54], pSVE-hRB^661W ^[DC unpublished data, 40], and pSVE-Rb^76t ^[41] were inserted to the BglII-HpaI sites of MSCV-GFP. Similarly, MSCV-Puro-Rb^WT ^was produced by inserting the pSVE-hRB^WT ^BamHI-StuI fragment in the MSCV-Puro BglII-HpaI site. MSCV-GFP-Rb^Δ21 ^was produced by replacing an MluI-MfeI fragment of MSCV-GFP-Rb^WT ^with the corresponding sequence from pGST-Rb^Δ21 ^[55].

BE-GFP was produced by replacing the EcoRV-SalI fragment of MA1, containing a PGK-TrkA cassette [56], with the polylinker sequence 5'-GGGGCTAGCTCTAGAACGCGTCGTACGACTCGAGTGTTTAAAC-3', and inserting an EF1αpromoter fragment between the polylinker NheI-XbaI sites. Rb^WT^, Rb^661W^, Rb^Δ21^, and Rb^76t ^cDNAs were transferred as BssHII-BsrGI fragments from MSCV-GFP to the BE-GFP MluI-BsiWI sites. p27^KIP1 ^cDNA was transferred as a KpnI-XbaI fragment from cDNA_3_-p27 (kindly provided by A. Koff) to the corresponding BE-GFP sites.

### Cell growth, virus production, infection, and Y79 sublines

Y79 cells were obtained from the ATCC and cultured in RPMI 1640, 10% fetal calf serum (FCS), penicillin, streptomycin, and L-glutamine, in a humidified 5% CO_2 _incubator. Retroviral supernatants used for direct infections were made by CaPO_4_-mediated co-transfection of Bing producer cells with the indicated MSCV-GFP constructs and pCL-Ampho [57]. Concentrated retrovirus was produced by CaPO_4_-mediated co-transfection of GP2 cells (Clontech) with MSCV-GFP constructs and pVSVg [58]. Lentivirus was produced using Lipofectamine 2000 (InVitrogen) to co-transfect 293T cells with the viral vector, pVSVg, and pΔ8.91 DNAs [59]. Cell supernatants were passed through 0.45 μm cellulose acetate filters 48 h after transfection. Virus was concentrated by centrifugation at 25,000 rpm for 90 min in a SW28 rotor, and suspended in 50 mM Tris, pH 7.8, 130 mM NaCl, and 1 mM EDTA. For infections, Y79 cells were plated at 2 × 10^6 ^cells per ml and combined with an equal volume of producer cell supernatants, or with up to 0.5 volume of concentrated virus, at 6 μg/ml polybrene. As MSCV-GFP titers were ~10-fold higher than for Rb derivatives, MSCV-GFP supernatants were diluted 10-fold prior to infection.

Cells transduced with MSCV-Puro or Rb derivatives were selected with 1.5 μg/ml puromycin for 11 days, and re-infected with MSCV-GFP and Rb derivatives 20 days after the initial infection.

### Analysis of infected cells

GFP expression and forward scatter were determined using a Becton-Dickenson FACSCaliber and CellQuest Software. Cell cycle positions of retrovirus-transduced cells were determined by fixing in 0.75% paraformaldehyde (PFA) for 30 min at 22°C, washing in PBS + 3% FCS, fixing in 70% ethanol at -20°C, staining in 0.05 mg/ml propidium iodide, 0.6% Nonidet-P40 in PBS, and 1 mg/ml RNAse A, and gating on diploid GFP+ cells. Cell cycle positions of lentivirus-transduced cells were determined as above but without PFA fixation or GFP gating.

For immunofluorescent detection of Rb, Y79 cells were attached to poly-L-lysine coated slides, fixed in 4% PFA for 10 min and stained with anti-Rb antibody G3-245 (1:200, Becton-Dickenson) and Cy3-conjugated donkey anti-mouse. For immunoblotting, lysates were separated and immunoblotted to Hybond-P and probed using ECL-Advance (Amersham), with mouse anti-Rb G3-245 (1:1,200), rabbit anti-p27 (1:150, SantaCruz sc-528), and rabbit anti-α-actin (1:1,000, Sigma A2066). To compare Rb expression after retroviral infection, infected cell lysates were combined with mock-infected cell lysates to normalize for the proportion of GFP+ cells.

*RB1 *cDNA expressed following retroviral transduction was detected by RT-PCR with forward primer 5'-GCTTGAGTTTGAAGAAACAGAAGAACC and reverse primer 5'-CTTTAGCTAATAAAAATGTGATCCAAGAAACTT, and with a GAPDH control.

### Gene expression analysis

RNA was prepared 60 h after lentivirus infection or after retrovirus infection followed by FACS enrichment to >97% GFP+ cells. In the latter case, cells were suspended in Buffer RLT (Qiagen) within 10 min after sorting. RNA was prepared using RNeasy (Qiagen) including on-column DNAse digestion, and RNA quality confirmed by Agilent BioAnalyzer. For microarray analysis, 10 ng of each RNA was used to prepare probes with the Ovation Biotin Amplification System (Novogen), and probing Affymetrix U133 Plus 2.0 GeneChip. For real-time quantitative PCR (RT-qPCR), oligo dT-primed cDNA was prepared from 750 ng RNA and ImProm-II reverse transcriptase (Promega), and cDNA transcribed from 5 ng of RNA subjected to RT-qPCR using iTaq SYBR Green Supermix (Bio-Rad), and normalized to β-actin RNA measured by Taqman (Applied Biosystems). PCR primers were for *cyclin E1 *(forward 5'-CGTGCGTTTGCTTTTACAGA, reverse 5'-AGCACCTTCCATAGCAGCAT); *cyclin E2 *(forward 5'-CCTCCATTGTGAGATAAGGACA, reverse 5'-GCCTATGTACAGCAAGTTTTCA); *Brn-2 *(forward 5'-CAGAGAGATGGCAAGCACTG, reverse 5'-TCAGGAAGCTGCATTTTGTG); and *HSP70A1B *(forward 5'-CCGAGAAGGACGAGTTTGAG, reverse 5'-GCAGCAAAGTCCTTGAGTCC).

## Competing interests

The author(s) declare that they have no competing interests.

## Authors' contributions

DC conceived the study, participated in its design, constructed lentiviral vectors, analyzed the effects of retroviral and lentiviral Rb transduction, and drafted the manuscript. ROF designed, constructed, and analyzed transduction by retroviral Rb vectors. DHA and TCL participated in the conception and design of the study. All authors read and approved the final manuscript.
